# Computational design of a symmetrical *β*-trefoil lectin with cancer cell binding activity

**DOI:** 10.1038/s41598-017-06332-7

**Published:** 2017-07-19

**Authors:** Daiki Terada, Arnout R. D. Voet, Hiroki Noguchi, Kenichi Kamata, Mio Ohki, Christine Addy, Yuki Fujii, Daiki Yamamoto, Yasuhiro Ozeki, Jeremy R. H. Tame, Kam Y. J. Zhang

**Affiliations:** 1 Graduate School of Medical Life Science, Yokohama City University, 1-7-29 Suehiro, Yokohama, Kanagawa 230-0045 Japan; 2 Structural Bioinformatics Team, Division of Structural and Synthetic Biology, Center for Life Science Technologies, RIKEN, 1-7-22 Suehiro, Tsurumi, Yokohama, Kanagawa 230-0045 Japan; 3 Laboratory of Biomolecular Modelling and Design, Department of Chemistry, KU Leuven, Celestijnenlaan 200G, 3001 Heverlee, Belgium; 4 Department of Pharmacy, Graduate School of Pharmaceutical Science, Nagasaki International University, 2825-7 Huis Ten Bosch, Sasebo, Nagasaki 859-3298 Japan; 5 Laboratory of Glycobiology and Marine Biochemistry, Graduate School of NanoBio Sciences, Yokohama City University, 22-2, Seto, Yokohama, Kanagawa 236-0027 Japan

## Abstract

Computational protein design has advanced very rapidly over the last decade, but there remain few examples of artificial proteins with direct medical applications. This study describes a new artificial *β*-trefoil lectin that recognises Burkitt’s lymphoma cells, and which was designed with the intention of finding a basis for novel cancer treatments or diagnostics. The new protein, called “Mitsuba”, is based on the structure of the natural shellfish lectin MytiLec-1, a member of a small lectin family that uses unique sequence motifs to bind *α*-D-galactose. The three subdomains of MytiLec-1 each carry one galactose binding site, and the 149-residue protein forms a tight dimer in solution. Mitsuba (meaning “three-leaf” in Japanese) was created by symmetry constraining the structure of a MytiLec-1 subunit, resulting in a 150-residue sequence that contains three identical tandem repeats. Mitsuba-1 was expressed and crystallised to confirm the X-ray structure matches the predicted model. Mitsuba-1 recognises cancer cells that express globotriose (Gal*α*(1,4)Gal*β*(1,4)Glc) on the surface, but the cytotoxicity is abolished.

## Introduction

In 2012, a lectin designated “MytiLec” was isolated from the Mediterranean mussel *Mytilus galloprovincialis*, and found to bind sugar chains with *α*-D-galactose at the reducing end^1^. The polypeptide chain has three well-conserved repeats of a roughly 50-residue sequence, and adopts a *β*-trefoil fold. Together with two other sea-mussel proteins, *Crenomytilus grayanus* lectin (CGL)^2^ and *Mytilus trossulus* lectin (MTL)^3^, MytiLec forms a small subfamily of closely related lectins with no sequence similarity to other proteins. They show bacteriostatic properties, and appear to play a role in innate immunity, alongside other shellfish lectins^4, 5^. DNA sequencing subsequently identified two related genes in *M. galloprovincialis*, encoding MytiLec-2 and MytiLec-3, which include a pore-forming aerolysin-like domain attached to the sugar binding domain^6^, and MytiLec was therefore renamed “MytiLec-1”. Globotriose (abbreviated Gb3), Gal*α*(1,4)Gal*β*(1,4)Glc, is a component of glycosphingolipids found on the surfaces of certain cancer cell types including Burkitt’s lymphoma^7^. MytiLec-1 shows cytotoxic effects towards such cells, but activity is dependent on sugar binding and blocked by the addition of an *α*-galactoside^1^. The mechanism of cytotoxicity is under investigation, but involves entry of the lectin into the cell and triggering of apoptosis^8^. MytiLec-1 is unusual among natural *β*-trefoil lectins in that each sequence repeat forms a sugar-binding site, so that each polypeptide binds three identical ligands^9^, whereas, in general, *β*-trefoil lectins bind only one ligand per protein subunit.

Lectins are a diverse group of carbohydrate binding proteins including very different overall architectures, such as *β*-sandwich, *β*-trefoil and *β*-propeller structures^10^. The affinity of individual binding sites for carbohydrate is rather weak, but a high avidity for larger substrates may be achieved through the combined action of multiple binding sites. For example, a hexavalent *β*-propeller neolectin was designed to bind glycolipids, and it was found that at least two binding sites are required for avidity. The distance between two adjacent binding sites strongly affects the ability of the protein to bend and invaginate membranes^11^. Although lectins are well-known for their medical properties, many are too toxic to be of clinical use. A number of different lectins have however been investigated as potential treatments for cancer^12^, including ABL, from the edible mushroom *Agaricus bisporus* ^13, 14^. ABL recognises the Thomsen-Friedenreich antigen (TF antigen), a well-known disaccharide cancer biomarker. Almost all the markers targetted by these lectins are *β*-linked, so the MytiLec family provides an orthogonal specificity for attacking particular cell types. MytiLec-1 is also highly unusual among cytotoxic lectins in having only a *β*-trefoil structure. Generally such a sugar-binding domain serves merely to bring another functional or toxic domain to the target cell, as appears to be the case for MytiLec-2 and MytiLec-3. The simplicity of structure makes MytiLec-1 an attractive template for the creation of an artificial symmetrical version, which could hopefully be later incorporated into a larger protein complex giving higher avidity for the target cells and more effective cell killing at lower doses. The *β*-trefoil fold is adopted by widely divergent sequences, and many models of such proteins are known. Almost 20 years ago it was suggested that all *β*-trefoils are descended from a common ancestor^15^, but a later analysis of 1167 non-redundant sequences showed that there are many cases of greater similarity between the subdomains of a given *β*-trefoil protein than between subdomains from different proteins^16^. This result implies that *β*-trefoils have largely evolved independently, from different duplication events, rather than descending from a universal ancestral domain.

Different groups have created symmetrical *β*-trefoil variants to help understand protein folding and evolution^16–19^. The group of Blaber used “top-down symmetric deconstruction” to impose perfect three-fold symmetry on sequences derived from fibroblast growth factor-1, by cycles of symmetrisation and stability screening^17^. The process yielded Symfoil-4P, which is significantly more stable than the parent protein but without its natural function. The group of Meiering adopted a different, more computational approach, using a template specifically chosen for having the highest sequence symmetry among natural trefoil proteins; the resulting structure, called Threefoil, is extremely stable and retains the sugar binding of the parent^16^. Like the great majority of computational protein designs, these proteins so far have found no medical or commercial application, but they demonstrate that duplication of sequence motifs within a single polypeptide chain, giving identical structural components to the folded protein, is by no means incompatible with thermostability. Recently, we have experimented with the creation of perfectly symmetrical proteins from natural templates based on the view that many nearly symmetrical ring-shaped proteins have evolved through exactly such an intermediate phase. We designed Pizza, a *β*-propeller protein with six identical blades, and showed it can fold readily and is extremely stable^20^. A key element of the design approach we adopted was to model the evolutionary development of the chosen natural template, and work from the most probable sequence that represented the blade of the presumed symmetrical intermediate^21^. Here we have adopted a similar procedure and applied it to MytiLec-1, to create a related protein with three identical subdomains, that retains sugar binding activity and the ability to bind selected cell types. MytiLec-1 is strongly stabilised by forming a tight dimer, and mutating the dimerisation interface yields unstable monomers^9^. Symmetrising the *β*-trefoil eliminated this interface to create a new monomeric form. We have refined the X-ray crystallographic structure of the symmetrical lectin to high resolution, and show that this artificial protein is significantly more stable than the parent protein, despite the loss of the dimer interface.

## Results

### Computational design

Crystal structures of MytiLec-1 (both with and without ligands) were previously refined to high resolution^9^, and the structure of the apo-protein (PDB 3WMU) was chosen as the template to create Mitsuba. The sub-domains of MytiLec-1 (labelled A, B and C from the N- to C-terminus) show more than 50% amino acid sequence similarity, and superposing these regions of the model with each other shows a main-chain root mean square deviation (RMSD) close to 1.0 Å. The sequences of the separate subdomains were structurally aligned, and ancestral sequence prediction (based on the alignment and the inferred phylogenetic tree) was carried out using the FastML server^22^. Symmetrical backbones were produced using Rosetta symmetric docking, using the three individual subdomains of MytiLec-1 as templates, but only subdomain-A gave the highest score to a trefoil-like assembly, so the other models were discarded. The three symmetrically-arranged copies of subdomain-A were concatenated into a triple repeat with Gly-Asp-Gly tripeptide linkers and the backbone energy minimised using MOE (Molecular Operating Environment, Chemical Computing Group, Montreal, Canada). The predicted ancestral sequences were mapped onto the symmetrised backbone model using PyRosetta^23, 24^, and each sequence was ranked by the Rosetta score. With only three related basis sequences to work with, only a limited region of sequence space could be sampled and the model scores did not show strongly favoured sequences. A broad spread of energy/RMSD scores was obtained, with the lowest energy model having a large deviation from the starting model, with a C*α* RMSD of 1.6 Å. This is partly because residues linking the subdomains of MytiLec-1 are also involved in the dimerisation interface, and the pseudo-symmetry of the natural protein is broken at this point. Furthermore the model showed a large central cavity lined by hydrophobic residues, which appeared unlikely in a stable protein structure. Comparison of the backbone model at this stage with the symmetrical trefoils Symfoil^18^ and Threefoil^16^ structures showed Threefoil to be more similar. Threefoil has a single tryptophan residue in each subdomain forming a hydrophobic core, so in an attempt to improve the core packing and stabilise the linker region, linker sequences (6 or 9 residues) of the Threefoil structure (PDB 3PG0)^16^ were grafted onto the backbone. Subsequent re-minimisation of the backbone model and fitting of MytiLec ancestral sequences to all positions except the Threefoil-derived linker gave designs with a smaller central cavity. A small number of sequences have energy scores somewhat lower than the bulk of the distribution (Supplementary Figure 1). The C*α* RMSD values for these more stable models were around 1.05 Å, a significant improvement on the first backbone template. The sequence with the lowest energy score was termed “Mitsuba-1”. This 143 residue sequence includes 6 residues of the Threefoil linker, shows 61% identity with MytiLec-1, and retains the HxDxH and HPxGG motifs crucially involved in binding galactose. The galactose binding sites were not explicitly preserved by manual restraint, but were retained throughout the modelling steps by the ancestral reconstruction. For comparison, the models with the smallest C*α* RMSD (“Mitsuba-2”) and the smallest internal cavity (“Mitsuba-3”) were also selected for expression. Both are derived from the backbone built with 9 residues of the Threefoil linker region, including the tryptophan residue.

### Protein expression and oligomeric structure

**Figure 1:**
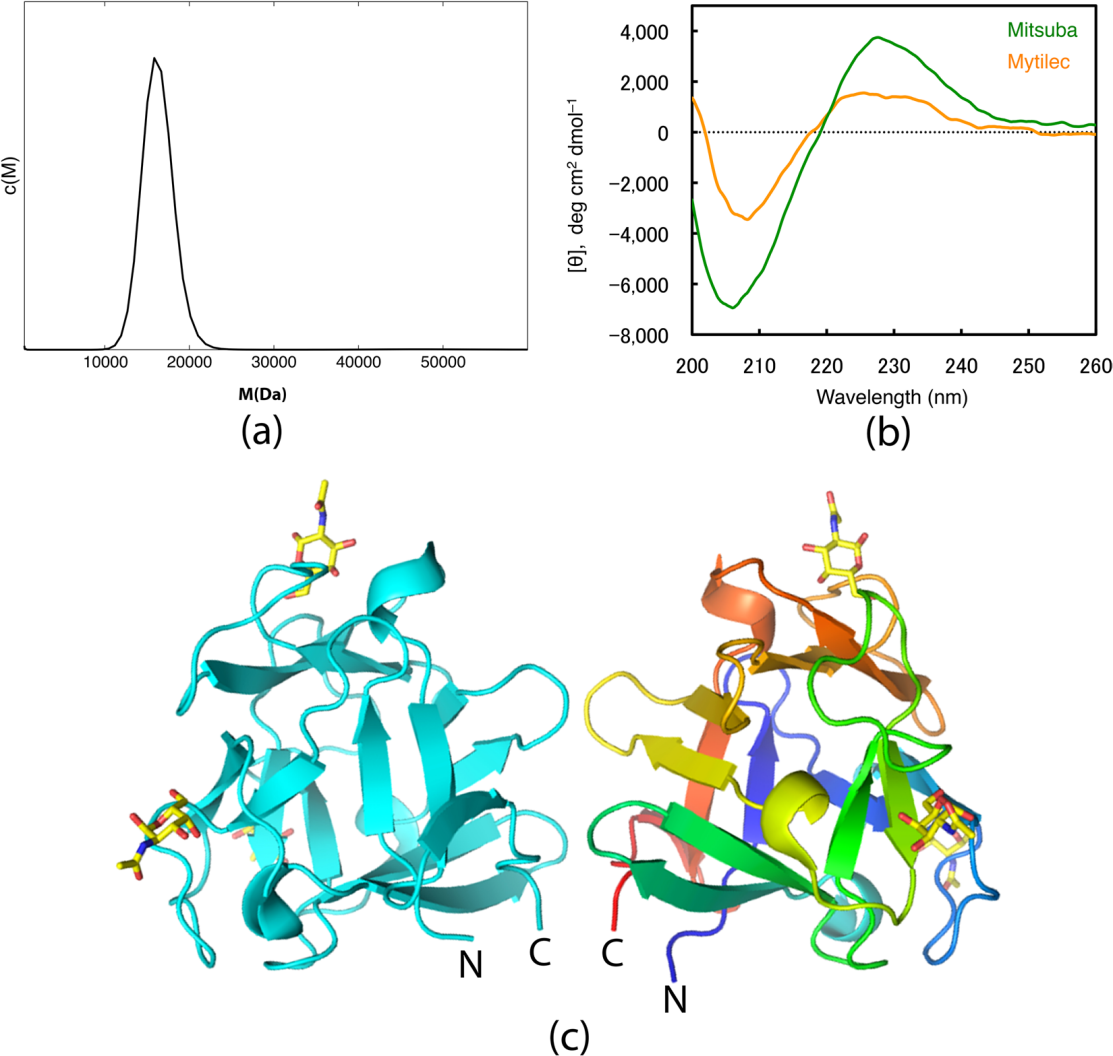
(**a**) Molecular weight determination by analytical ultracentrifugation. Sedimentation velocity data were processed to reveal relative abundance c(M) of species with molecular weight M ranging up to 100 kDa. The plot shows the curve for M values from 500 Da to 60 kDa. No species were present other than monomer, with a predicted M of 16553 Da. (**b**) The circular dichroism of Mitsuba-1 (green) and MytiLec-1 (orange) compared. Both models show similar features expected of a structure containing *β*-sheet, but these are more pronounced for Mitsuba-1. (**c**) A ribbon diagram of MytiLec-1 (PDB 3WMV), showing both subunits of the dimer, one coloured cyan and the other from blue (N terminus) to red (C terminus). N-acetylgalactosamine ligands are shown as sticks, with carbon atoms coloured yellow, oxygen red and nitrogen blue.

A DNA coding sequence was designed for each chosen protein by backtranslating with an in-house program. Codon usage was optimised for expression in *E. coli* and the synthesised genes were inserted into the standard expression vector pET28, allowing the protein to be expressed and purified using a thrombin cleavable histidine tag. Mitsuba-1 expressed to a level similar to MytiLec-1, and could be concentrated to 10 mg/mL, indicating that it is correctly folded and stable. In contrast, the expression levels of Mitsuba-2 and Mitsuba-3 were very low, less than 0.1 mg per litre of culture, and no experimental tests of these proteins could be performed. The sequences of the three designed proteins are compared in Supplementary Figure 2, showing that Mitsuba-2 and Mitsuba-3 contain a tryptophan residue equivalent to that of Threefoil, but Mitsuba-1 retains the phenylalanine of earlier models in this position. Analytical ultracentrifugation (AUC) shows that Mitsuba-1 is a monomer in solution, with no indication of larger species or aggregation (Fig. 1A), a result confirmed by size-exclusion column chromatography (Supplementary Figure 3). Circular dichroism indicated that the protein adopted a stable fold, rich in *β* structure (Fig. 1B), allowing the melting temperature to be determined to be 55 °C (Supplementary Figure 4A). Unfolding experiments were also carried out by observing the change in circular dichroism at 228 nm with added increments of guanidinium hydrochloride (Supplementary Figure 4B). Fitting the curves obtained to a two-state model yielded denaturation mid-points at GdnHCl concentrations of 3.6 M and 1.3 M for Mitsuba-1 and MytiLec-1 respectively. MytiLec-1 is a tightly-bound dimer, as shown in Fig. 1C, but the interface of the natural protein is not preserved in Mitsuba-1.

### Crystallographic analysis

**Figure 2:**
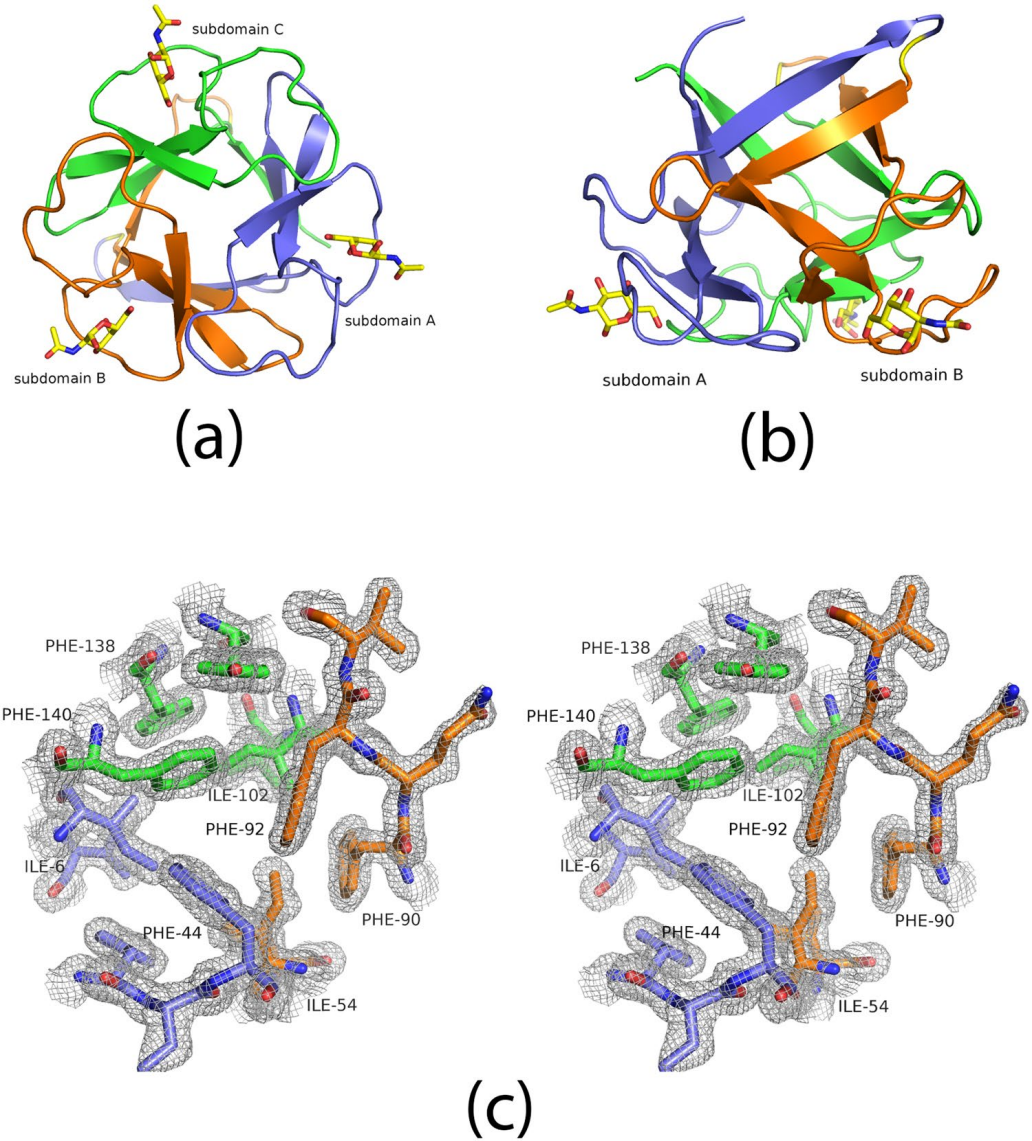
The overall structure of Mitsuba-1. (**a**) The C *α* trace of Mitsuba-1, looking along the pseudo-three-fold symmetry axis. The trace is coloured by subdomain, with *α*-helices shown as coils and *β*-strands as arrows. *α*-GalNAc ligands are shown as sticks with yellow carbon atoms. The subdomains are coloured purple, orange and green from N to C terminus. Structural figures were drawn using PYMOL^54^. Secondary structure was determined automatically. (**b**) A view of the model shown but with the three-fold symmetry axis vertical. (**c**) The 2mFo-DFc electron density map, shown in stereo, contoured at 1 *σ*, covering a selection of residues near the symmetry axis.

**Figure 3:**
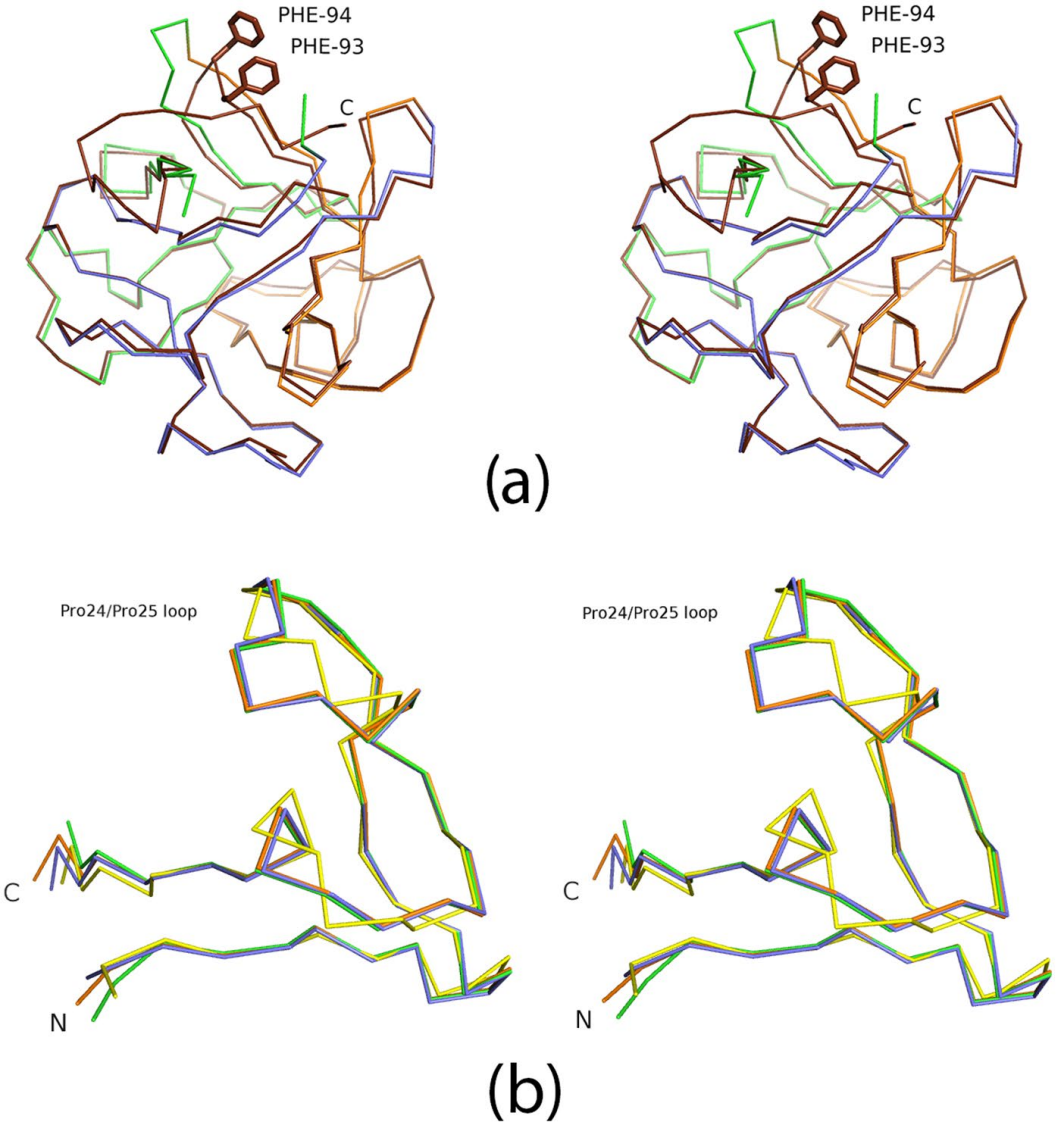
The subdomain structure of Mitsuba-1. (**a**) Stereo view of MytiLec-1 C*α* trace (chocolate brown) overlaid onto Mitsuba-1 (coloured by subdomain as in Fig. 2). Phe 93 and Phe 94 of MytiLec-1 are shown as sticks, indicating that the surface loop of the protein at this point is truncated relative to other subdomains. (**b**) Stereo overlay of the individual subdomains of Mitsuba-1 and a single subdomain of Threefoil (shown in yellow). Differences between Mitsuba-1 and Threefoil are pronounced at the loop including Pro 24 and Pro 25, or equivalent residues.

The complex of Mitsuba-1 and NAcGal crystallised in space-group *P*2_1_, and diffracted to 1.54 Å resolution. Preliminary phases could be determined directly by molecular replacement with the designed model. Crystallographic data and refinement statistics are given in Table 1. One complete monomer is found in the asymmetric unit, with no breaks in the the 2mFo-DFc electron density map of the chain, and there are no Ramachandran outliers. A histidine residue, from the linker peptide after cleavage of the His-tag, is visibly attached to the methionine residue at the N-terminus of the designed sequence. The structure is well ordered with each subdomain forming a *β*-sheet of four strands (Fig. 2). Overlaying 143 C*α* atoms of Mitsuba-1 and MytiLec-1 gives an RMSD value of 0.86 Å, showing the structures are highly related. Side-chains of Mitsuba-1 and MytiLec-1 show considerably poorer overlap than the main-chain atoms, not only among the buried residues but also at surface loops. Deviations of the chain trace are mainly seen close to the dimer interface of MytiLec-1. Two copies of MytiLec-1 associate by close interaction through a highly apolar interface formed by two adjacent phenylalanine residues^9^, but these side-chains (F93 and F94) are not preserved in Mitsuba-1 since they are found near a loop of subdomain B which is truncated compared to the other subdomains of the natural protein (Fig. 3A). Conversely residues 139 to 149 (the C terminus) of MytiLec-1 pass across the symmetry axis of the trefoil and have no counterpart in Mitsuba-1, which is therefore six residues shorter overall. Comparing the sub-domains within Mitsuba-1 shows that residues 1–46, 49–94 and 97–142 overlay closely, but the chain termini, especially the N-terminus causes some differences (Fig. 3B). The RMSD values from superposition of the 46 C*α* atoms in each of the subdomains A and B, A and C, and B and C, are 0.91 Å, 1.02 Å, and 0.31 Å respectively. The three-fold symmetry prevents internal residues of Mitsuba-1 from approaching the symmetry axis too closely, and a central cavity is found in the structure with a volume close to 100 Å^3^ according to KVFinder^25^. MytiLec-1 has a smaller cavity with a volume of about 40 Å^3^.

**Table 1:** ^*a*^Rmerge = ∑*I*_*i*_ − 〈*I*〉/∑*I*_*i*_, where *I*_*i*_ is the intensity of an observation and 〈*I*〉 is the mean value for this reflection, and the summations are over all reflections.

Data-set	Mitsuba-1
Data collection statistics	
Space group	*P*2_1_
Wavelength (Å)	0.980
Unit cell (Å)	a = 42.78, b = 38.68 c = 42.76, *β* = 97.56°
Resolution range (overall/outer shell)	50.0–1.54/1.58–1.54
Reflections (measured/unique)	195585/57287
Completeness (overall/outer shell, %)	96.0/89.6
^*a*^R_*merge*_ (overall/outer shell, %)	6.3/21.5
Multiplicity (overall)	2.9/2.1
Average *I*/*σ*(*I*) (overall/outer shell)	29.2/4.6
Refinement statistics	
Resolution range (Å)	42.4–1.54
^*b*^R-factor/free R-factor (%)	15.0/19.7
Rmsd bond lengths (Å)/angles (°)	0.022/2.19
No. water molecules	163
Average B factors (Å^2^) (protein/water/ligand)	13.7/27.5/14.3
% residues with favoured Ramachandran angles	97.2
% residues with acceptable Ramachandran angles	2.8
% residues with outlier Ramachandran angles	0

Values in parentheses are for the highest resolution shell. ^*b*^R factor is ∑_*h*_||*Fo*(*h*)| − |*Fc*(*h*)||/∑_*h*_ *Fo*(*h*), where *Fo* and *Fc* are the observed and calculated structure factor amplitudes, respectively. The free R factor was calculated with 5% of reflections omitted from the refinement.

**Figure 4:**
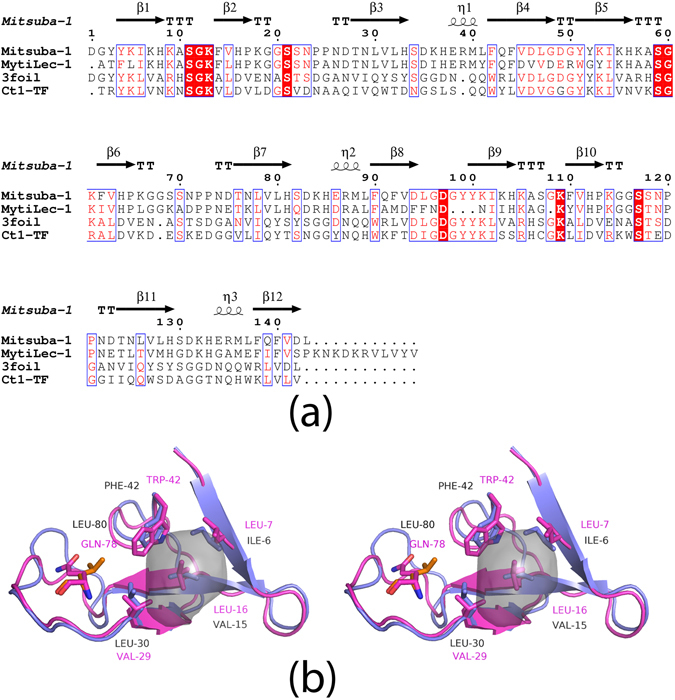
Comparison of Mitsuba with Threefoil. (**a**) Sequence alignment of Mitsuba-1 with related *β*-trefoils. The secondary structure elements of Mitsuba-1 (detected automatically) are shown as arrows and coils. The PDB entries for Threefoil and Ct1 are 3PG0 and 3VSF respectively. The N-terminal catalytic domain of Ct1 is omitted. Mitsuba-1 shows 29% sequence identity to Threefoil, and only 22% to Ct1. Threefoil shows 48% sequence identity with the Ct1 trefoil domain. The figure was drawn using ESPRIPT^58^. (**b**) A stereo ribbon diagram of the first subdomain of Mitsuba-1, shown in purple. The central cavity of the protein is shown as a translucent grey surface. Threefoil (shown in pink) has several mutations compared to Mitsuba-1 in the central region, and the notable mutations are shown as sticks and labelled. Threefoil has Trp 42 (and two equivalents in the other subdomains) in place of Phe 42 of Mitsuba-1. This larger side-chain is accommodated by Gln 78 and the altered backbone structure nearby, but Leu 80 of Mitsuba-1 would clash with the tryptophan. The hydrophobic core of Threefoil is also filled by Leu 16; replacements at positions 7 and 29 on either side of this side-chain allow better packing, leaving no significant cavity. Cavity analysis was performed with KVFinder^25^.

A direct comparison of the Mitsuba-1 structure with the entire PDB was carried out with DALI^26^. Unsurprisingly, the top hits are models of MytiLec^9^ and CGL^27, 28^ (for example PDB models 3WMV and 5DUY), sharing a Z-score of 27.2, and a number of *β*-trefoil proteins are detected. Less expected was that the Threefoil model, with a Z-score of 23.5, ranked slightly behind Ct1, an exo-beta-1,3-galactanase from *Clostridium thermocellum*. Ct1 is a glycoside hydrolase that uses a non-catalytic *β*-trefoil domain to help bind substrate, and models of this protein include PDB 3VSF^29^. A comparison of Mitsuba-1 with related sequences is shown in Fig. 4. Superposing the Mitsuba-1 and Threefoil models shows that 122 C*α* atoms can be overlaid with an RMSD of 1.22 Å. Threefoil has no detectable central cavity, in keeping with its high stability^16^, largely due to the presence of a tryptophan residue in place of Phe 42 of Mitsuba-1. This tryptophan reside is also present in the sequences of Mitsuba-2 and Mitsuba-3, as mentioned above, but neither of these sequences could be expressed and purified. A comparison of the central regions of Mitsuba-1 and Threefoil is shown in Fig. 4B, showing that a number of internal mutations and a shift of the backbone create space for the tryptophan side-chain in the latter protein.

### Sugar binding sites

**Figure 5:**
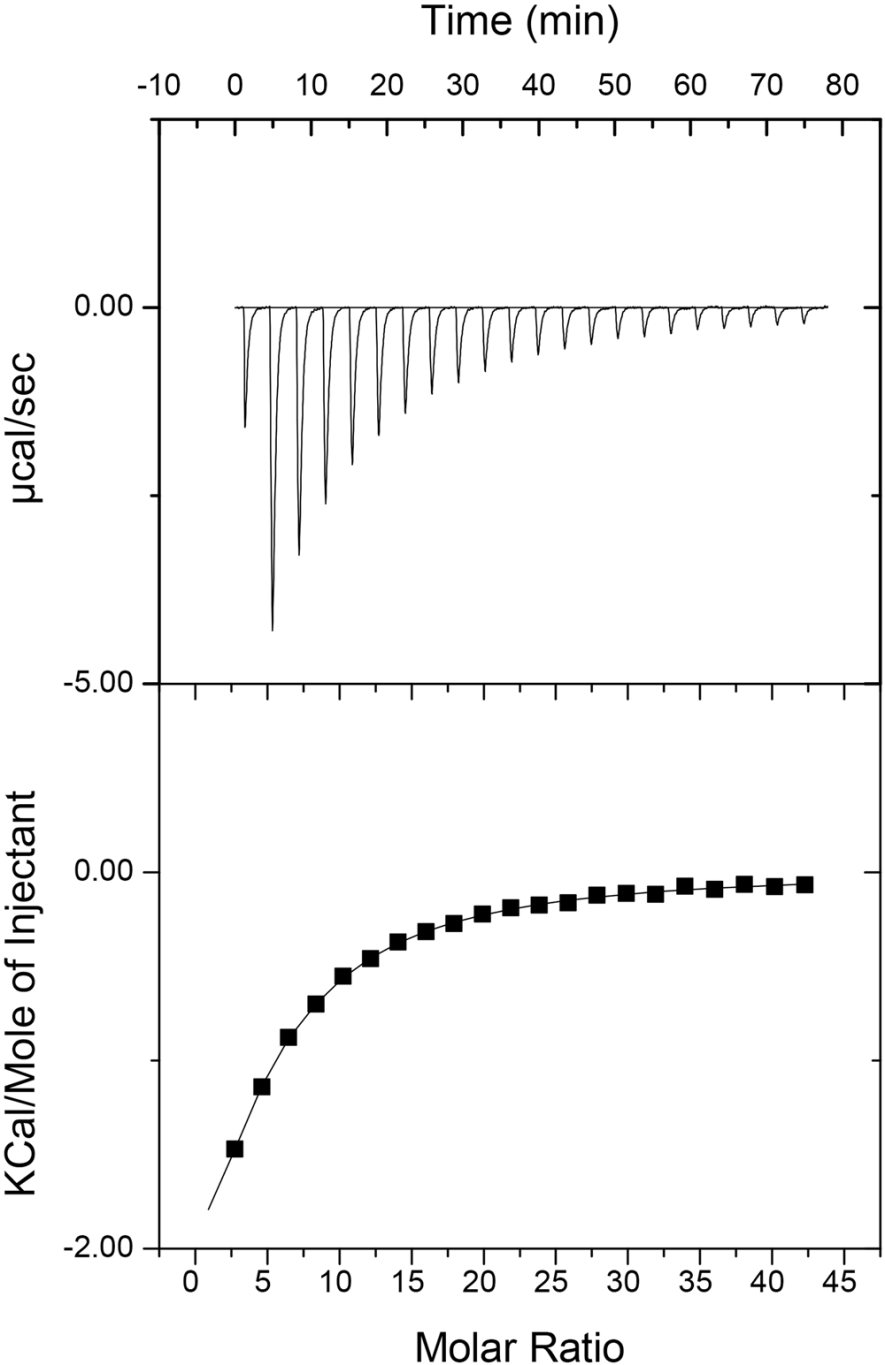
Isothermal titration calorimetric determination of the affinity of Mitsuba-1 for N-acetyl galactosamine. Fitting to a single-site model with stoichiometry of three sugar ligands to one protein molecule yields a *K*_*d*_ value of 0.33 mM. Binding is modestly exothermic under the conditions used, with Δ*H* of −6.5 kcal/mol, but weakened by the entropy change of −5.8 cal/mol/K.

Three GalNAc ligands are found at shallow binding sites related by the three-fold symmetry of the protein. The mode of sugar binding is common to MytiLec-1 and CGL^27, 28^. The contacts between Mitsuba-1 with GalNAc include five hydrogen bonds, including hydrogen bonds with two histidines and two aspartate residues. The HxDxH motif found at each binding site of MytiLec-1 is preserved, so that His 33, His 81 and His 129 of Mitsuba-1 form van der Waals contacts with the ligands but make no hydrogen bond with them. The Mitsuba-1 model, like MytiLec, shows no evidence of a significant role for water at any of the three sites in the asymmetric unit^9^. Each sugar ligand is well-ordered in the electron density map determined for Mitsuba-1 (Supplementary Figure 5), suggesting tight binding, but from earlier work with MytiLec^9^ and CGL^28, 30^ it is known that each binding site alone has rather weak affinity, and the avidity of the protein for cell surfaces displaying suitable sugar ligands arises from the multiplicity of sites. Using calorimetry, Mitsuba-1 was found to bind N-acetylgalactosamine with a *K*_*d*_ of 0.33 mM (Fig. 5). This is a slightly lower affinity than that found for MytiLec-1, despite the sequence conservation of the residues in direct contact with the ligand, suggesting that the second-shell residues in Mitsuba-1 may have contributed to the decrease in ligand binding affinity. There was no attempt made at optimising the ligand binding affinity in Mitsuba-1 during the design.

### Cytotoxicity and haemagglutination activity of Mitsuba-1

**Figure 6:**
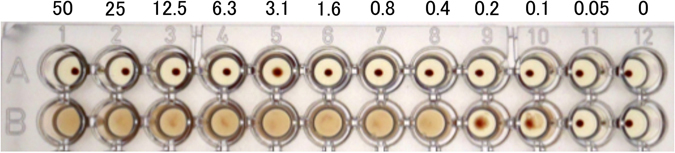
Haemagglutination assay. Lectin concentration is shown in *μ*g/mL. Mitsuba-1 (top row) showed no lytic effect on the red cells at any concentration tested, up to 50 *μ*g/mL. MytiLec-1 (bottom row) showed agglutination at concentrations down to 0.1~0.2 *μ*g/mL.

**Figure 7:**
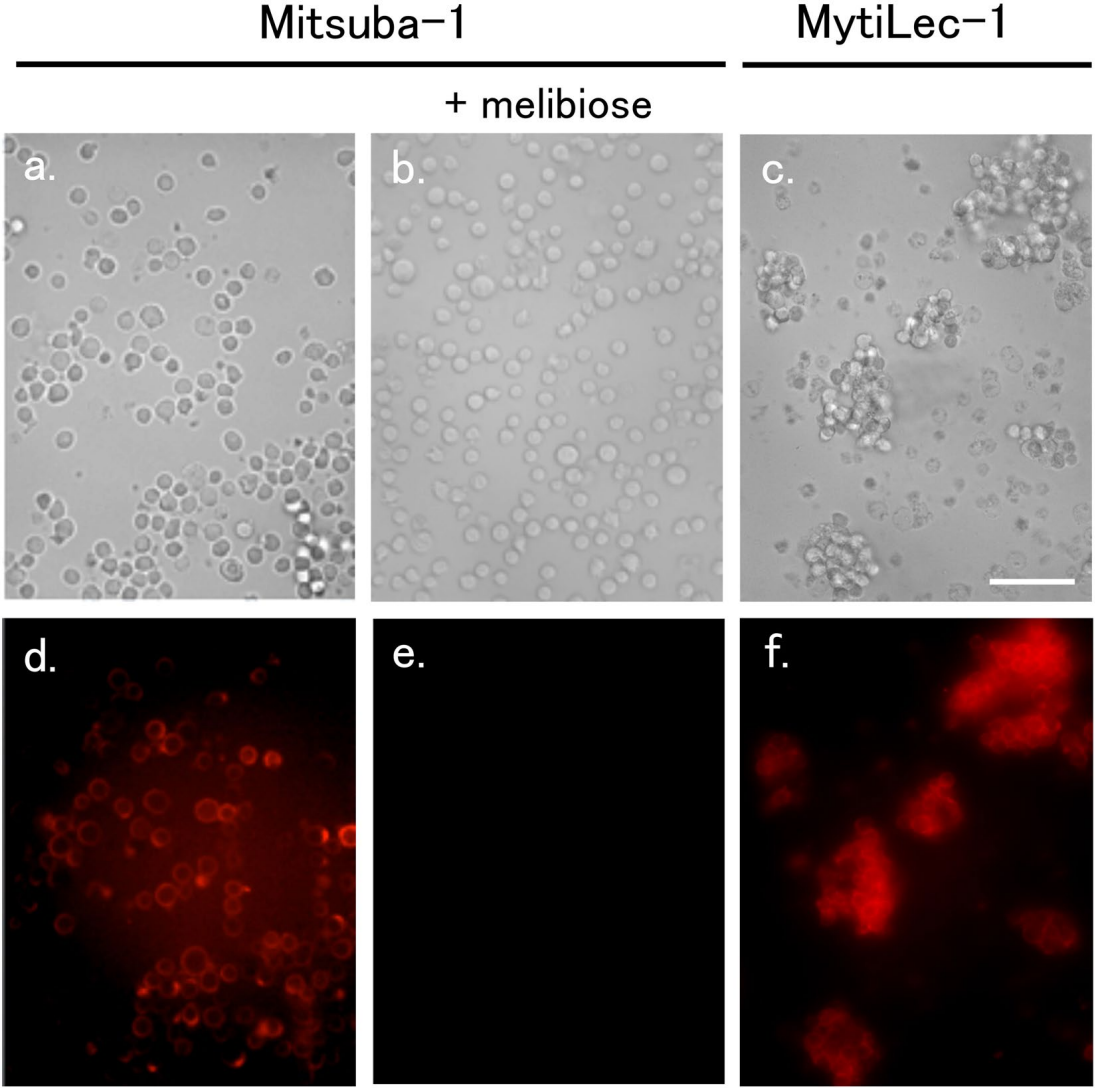
Agglutination of Raji cells. Mitsuba-1 (100 *μ*g/mL) showed no effect on cell structure in the absence (**a**) or presence (**b**) of melibiose. MytiLec-1 at the same concentration produced cell clumping (**c**). Labelled Mitsuba-1 binds to Raji cells in the absence (**d**) but not presence (**e**) of melibiose. Labelled MytiLec-1 is also found bound to the cell membranes (**f**).

MytiLec-1 shows strong haemagglutination activity, even at 0.1 *μ*g/L, but Mitsuba-1 showed no such activity at any concentration tested (Fig. 6). To determine if the lack of any apparent effect on red cells is due to a failure of Mitsuba-1 to bind the cell surface, the protein was labelled with a fluorescent tag (HyLite 555) and incubated with Raji cells, which are derived from Burkitt’s lymphoma. Mitsuba-1 failed to agglutinate Raji cells (Fig. 7A), unlike MytiLec-1 (Fig. 7C). Both Mitsuba-1 and MytiLec-1 were observed to bind (Fig. 7D,F). Binding of Mitsuba-1 was specifically inhibited by the presence of 20 mM melibiose (Gal *α*(1–6)Glc) (Fig. 7E). These results suggest that Mitsuba-1 may be able to select target cancer cells without haemagglutination of a patient’s red blood cells.

**Figure 8:**
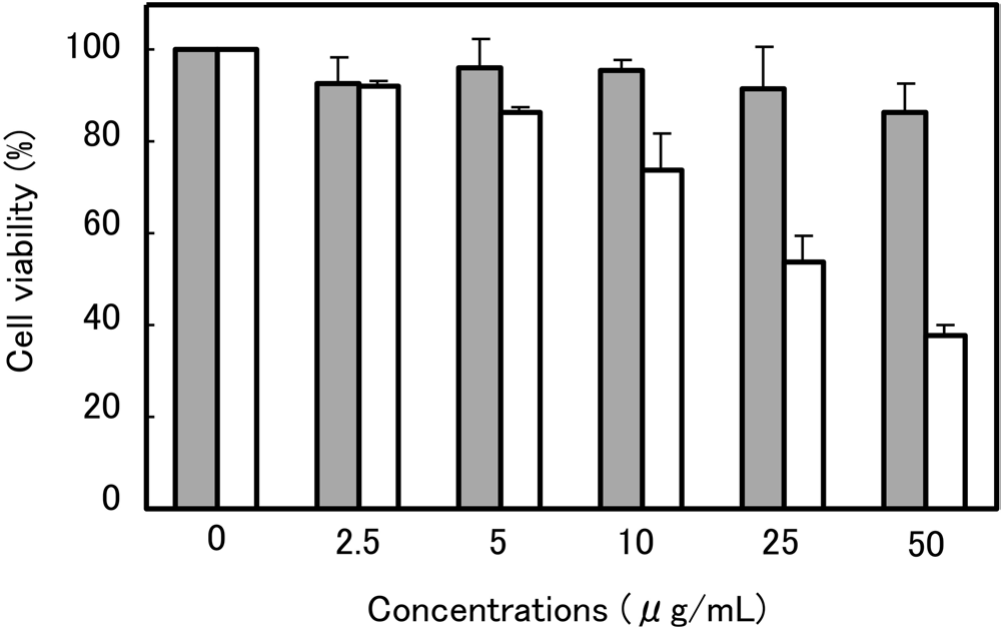
Cytotoxicity assay. MytiLec-1 and Mitsuba-1 were applied to Raji cells at concentrations up to 50 *μ*g/mL, and cell viability assessed after 48 h incubation at 37 °C. MytiLec-1 (white bars) showed a marked effect on cell survival rate, but Mitsuba-1 (grey bars) gives no significant drop in viability.

Mitsuba-1 (50 *μ*g/mL) is not found to reduce the viability of Raji cells, unlike MytiLec-1 (Fig. 8). This suggests that the dimeric form may be required for lectin-mediated cytotoxicity. Interactions with Gb3 have been reported to influence various signalling pathways^31–33^, but galactose binding alone is apparently insufficient to trigger apoptosis in Raji cells.

## Discussion

The *β*-trefoil is a common fold, with over 8000 sequences known or predicted to adopt such a structure. Automatic fold assignment by Pfam^34^ or SMART^35^ fails to categorise MytiLec-1 correctly, apparently because there is so much sequence variation among *β*-trefoil proteins, and MytiLec-1 forms a distinct subfamily with related mussel proteins. *β*-trefoil lectins are called R-type (ricin-like) carbohydrate recognition domains (CRDs), and they are found either as domains or free proteins. Within the CAZy classification scheme, these proteins are referred to as the carbohydrate-binding module (CBM) 13 family^36^. Cytotoxic lectins generally, like ricin, carry a non-lectin domain responsible for cell death^37, 38^, but several R-type lectins are known to directly affect the target cell, with no accessory domains required^39, 40^. MytiLec-1 is one of this group, and acts by entering sensitive cells and triggering apoptosis, but the mechanism remains poorly understood^8^. Previously we have created a monomeric form of MytiLec by substituting polar groups in place of the pair of phenylalanine side-chains at the dimer interface of the natural protein^9^. This MytiLec-F93DF94S mutant showed weak cytotoxicity, suggesting that the dimeric form of MytiLec-1 is important for eliciting an apoptotic response from cells. Binding to cell surfaces is expected to be weaker due to the halved number of sugar binding sites per protein molecule, but the amino acid residues at the binding sites are unchanged. Direct measurement of the binding of simple ligands to the monomer mutant by ITC proved impossible however since the protein was too insoluble^9^. Whereas MytiLec-F93DF94S proved too unstable to allow storage unfrozen for more than a few days, Mitsuba-1 appears to be stable for several weeks in storage at 4 °C without aggregation or proteolytic degradation. This allowed us not only to test the cytotoxicity of the protein but also to measure its biophysical properties such as unfolding temperature. Unfortunately the improvement in stability of Mitsuba-1 over MytiLec-F93DF94S is not accompanied by any increase in anti-cancer activity, so that the protein itself offers little hope of becoming a therapeutic agent, although it may be a means of directing other proteins or drugs to selected cell types.

Mitsuba-1 is a further test-case for the method of designing stable proteins with *C*_*n*_ symmetry by examining probable evolutionary routes to existing natural proteins. The reconstruction of ancestral protein forms - “molecular archaeology” - has been used for many years to examine protein evolution (see the review by Ogawa and Shirai^41^), and ancestral reconstruction was demonstrated by us several years ago to help design a thermostable propeller protein directly, with no experimental optimisation required^20^. It has also been used to create a pentameric tachylectin that retains the sugar-binding properties of the original template, but the authors of that study also used exhaustive library-based approaches in order to achieve the final result^42^. Several groups have noted the apparent stability of evolutionary intermediates^43–45^, and homologous sequence comparisons have now successfully been used to improve the stability of a variety of proteins for bacterial expression. Notably however, the method described by Goldenzweig and colleagues requires “several dozen homologues”, which indicates the variation required in sequence analysis to find probably favourable mutations^46^. Often a small number of residue replacements are all that is required to improve dramatically the stability of a protein, but finding these is not aided by comparing a very limited set of sequences. We have tested the ancestral reconstruction method by attempting to create a perfectly three-fold symmetrical *β*-trefoil from a template with limited internal symmetry. When using only the three subdomain sequences from MytiLec-1, no model was found with a sufficiently low predicted energy to justify experimental validation. Grafting six residues from the subdomain from Threefoil, an artificial *β*-trefoil with three tandem repeats, into the evolutionary reconstruction produced a novel sequence with an acceptable energy score. Expression of this sequence yielded a stable soluble protein, which readily crystallised and permitted a high-resolution structure to be refined.

Threefoil itself was designed from a natural protein whose three subdomains show 26 residues out of 47 to be identical. A further 5 residues were selected by comparison of 13 highly homologous structures, and the remainder were chosen as the most frequently observed in 10,000 models generated with Rosetta^16^. MytiLec-1 is a much less symmetrical protein with only 16 residues preserved in all subdomains, and a truncation of the central subdomain which helps create the dimerisation interface. The failure to determine a stable monomer by simple ancestral reconstruction in this case is therefore understandable from the restricted input sequences and their adaptation to different interactions. Protein structures depend on a relatively small number of anchoring side-chains to determine the fold^47^, and if these can be identified from simple alignments then the volume of sequence space to be searched is hugely reduced. Wide variation in sequences adopting a common fold not only helps highlight these anchor residues, but is also required to avoid in-breeding in ancestral reconstruction. To derive a symmetrical monomer from MytiLec-1 was therefore a challenge, and finally relied on a previous design, but our design strategy nevertheless produced a protein which is still much more related to MytiLec-1 than Threefoil (with sequence identities of 61% and 28% respectively). Ancestral reconstruction therefore is capable of producing functional symmetrical proteins, without any randomising steps or construction of libraries, provided that the initial sequence alignment provides sufficient sampling of sequence space.

The reported structure of Mitsuba-1 shows greatly improved properties over the monomeric MytiLec-F93DF94S mutant that was produced by simply replacing apolar residues at the dimer interface with polar ones. The backbone design however was complicated by the asymmetry of the parent structure, which itself has a considerable central cavity and is apparently strongly stabilised by dimerisation. The cavity size is significantly increased in Mitsuba-1, and could not easily be filled by simple mutations. Closely-related sequences with Phe 42 replaced by tryptophan proved too unstable to purify. Mitsuba-1 is clearly much more stable than MytiLec-1 in monomeric form despite the larger cavity, due to improved interactions throughout the structure. It may well prove possible to create an even more stable protein by simply grafting the ligand binding sites of MytiLec-1 onto Threefoil, but our purpose was to test the ancestral reconstruction method with the least human intervention possible rather than simply mutate a known structure. Notably however, simply adding more residues from Threefoil to the design did not yield more stable proteins. The central cavity in the protein is too small to be useful as a cargo hold, but the high stability of Mitsuba-1 makes it a promising protein for the development of novel diagnostic or therapeutic agents targetting a significant subset of cancer types.

## Methods

### Design

Backbone models were produced using Rosetta Symmetric Docking^24^, working from the crystal structure of MytiLec-1 (PDB 3WMV). Backbone energy minimisation and subdomain linking were carried out with MOE. 2000 possible ancestral sequences were predicted by the FastML server^22^, and mapped onto each symmetrical backbone model with Rosetta. Three different backbone structures were used for modelling with these sequences, one built from the MytiLec-1 subdomain A alone, and two others incorporating either 6 or 9 residues from Threefoil in each subdomain. The backbone using 6 Threefoil residues gave models with the best energy scores, including Mitsuba-1, the overall top scoring solution.

### Cloning

A synthetic gene encoding Mitsuba-1 was designed using in-house software with flanking NdeI and Xho1 restriction sites. Codon usage was optimised for expression in *E. coli* and any self-annealing sequences were corrected by silent mutagenesis. Three subdomains with identical sequence, 47 residues long, are linked by glycine residues (Gly 48 and Gly 96), giving a total length of 143 residues. The initiator methionine residue is numbered zero. The designed DNA sequence was excised from the supplied plasmid DNA and inserted into appropriately cut pET28, using T4 DNA ligase (Wako) at room temperature for 1 h. The ligation mixture was used to transform *E. coli* DH5 *α*, and pET28b-Mitsuba-1 was prepared using standard protocols. This vector directs expression of Mitsuba-1 carrying a thrombin-cleavable hexa-histidine tag at the N-terminus. The final purified protein product, after tag removal, has a sequence beginning with GSHMDG.

### Expression and purification

pET28b-Mitsuba-1 was transformed into *E. coli* BL21(DE3) pLysS, and cells were grown at 310 K with shaking in 6 L LB medium containing kanamycin and chloramphenicol (20 *μ*g ml^−1^). When the O.D. 600 of the culture reached 0.6~0.7, Mitsuba-1 expression was induced by adding IPTG to a final concentration of 0.2 mM, and growth was continued overnight at 293 K. The cells were collected by centrifugation at 3000 × g at 277 K for 30 min. The pellet was suspended in 100 mM Tris HCl pH 8.0/0.15 M NaCl/20 mM imidazole and then lysed by sonication on ice. The lysate was centrifuged at 38,000 × g at 277 K for 50 min. The supernatant solution was loaded onto a 5 ml volume nickel-sepharose column (GE Healthcare) equilibrated with 100 mM Tris HCl pH 8.0, 0.15 M NaCl, 20 mM imidazole, and after washing, eluted with 100 mM Tris HCl pH 8.0, 250 mM imidazole, 150 mM NaCl. The major protein fractions were collected and digested with thrombin overnight at 277 K during dialysis into 20 mM Tris HCl pH 8.0/100 mM NaCl. The protein was re-loaded onto the washed nickel-sepharose column and eluted with 20 mM Tris HCl pH 8.0/100 mM NaCl. The pooled fractions containing Mitsuba-1 were dialysed into 20 mM Tris HCl pH 7.4/20 mM NaCl before loading onto an SP-sepharose column (GE) equilibrated with the same buffer, and eluted with a gradient to 1 M NaCl. The pooled protein fractions were concentrated to 9 mg/ml using Amicon centrifugal filter units (Millipore). MytiLec-1 was expressed and purified as described previously^9^.

### Circular dichroism spectroscopy

CD spectra were measured using a JASCO J-1500 spectrometer with 0.1 mg/mL protein in 10 mM HEPES pH 7.4 and 100 mM NaCl, placed in a 1 mm path-length quartz cell. Chemical denaturation with guanidinium hydrochloride was carried out using 0.3 mg/mL protein samples. The process was monitored at 228 nm in steps of 0.25 M GdnHCl. The denaturation curve was fitted to a two-state model using the Marquardt-Levenberg algorithm. Thermal denaturation was also monitored at 228 nm, using temperature steps of 0.2 K. 0.25 mg/mL protein samples were held in a 2-mm path-length quartz cell with a screw lid. The data were fitted to a two-state model (folded/unfolded) for the Mitsuba-1 protein, and a three-state model (folded, dissociated, unfolded) for the Mytilec-1 dimer.

### Analytical Ultracentrifugation

The sample concentration was estimated as 1.0 *μ*g ml^−1^ from absorbance at 280 nm. Sedimentation velocity experiments were carried out using an Optima XL-I analytical ultracentrifuge (Beckman-Coulter) using an An-50 Ti rotor. Cells with a standard Epon two-channel centre-piece and sapphire windows were used. 400 *μ*L of the sample and 420 *μ*L of the reference solution (50 mM potassium phosphate pH 7.4 and 0.1 M NaCl) were loaded into the cell. The rotor was kept stationary at 293 K in the vacuum chamber for 1 h prior to each run for temperature equilibration. Absorbance at 280 nm scans were collected at 10 min. intervals during sedimentation at 50,000 rpm. The resulting scans were analysed using the continuous distribution *c*(*s*) analysis module in the program SEDFIT^48^. Frictional ratio (f/fo) was allowed to float during fitting. The *c*(*s*) distribution was converted into a molar mass distribution *c*(*M*). Partial specific volume of the protein, solvent density, and solvent viscosity were calculated from standard tables using the program SEDNTERP^49^.

### Crystallisation and structure determination

Co-crystals of Mitsuba-1 were grown using 9 mg/ml protein with 5 mM N-acetyl-D-galactosamine. Crystallisation experiments were performed at 293 K using the hanging-drop vapor diffusion method. Crystals grew in 25% (w/v) PEG 6000, 0.1 M MES pH 6.5. Crystals were washed briefly in mother liquor containing 18.5% glycerol as cryo-protectant before being stored in liquid nitrogen. Data were collected at beam-line 1 A of the Photon Factory, Tsukuba, using incident radiation of 0.98 Å wavelength. A total of 250 images of 1° oscillation were collected for the native dataset. Data processing and scaling were carried out with HKL2000 and SCALEPACK^50^. The space-group was found to be *P*2_1_, with one molecule in the asymmetric unit. Data statistics are given in Table 1. An initial model was created using molecular replacement, starting with PDB 3WMV as a search model. Manual modifications were carried out with COOT^51^. Refinement was carried out with REFMAC^52^ and the CCP4 suite^53^. TLS group refinement was not used. The Ramachandran plot of the native model shows no residues in unusual positions. Isotropic temperature factors were refined with default isotropic restraints giving an R-factor close to 15%. Water molecules were checked manually for steric clashes or unusually shaped electron density; several were fitted with partial occupancy. Figures were prepared with PYMOL^54^. Data collection and refinement statistics are shown in Table 1.

### Haemagglutination activity assays of Mitsuba-1 and MytiLec-1

Haemagglutination assays were performed in 96-well U-shape plates as described previously^55^. 20 *μ*L of a 2-fold dilution of each protein (20 mg/mL starting concentration) in TBS was mixed with 20 *μ*L of a 1% suspension (with TBS; v/v) of trypsinised and glutaraldehyde-fixed rabbit erythrocytes that was washed with saline. The plate was incubated at room temperature for 1 h, and the formation of a sheet (agglutination-positive) or dot (agglutination-negative) was observed and scored against the lectin titre.

### Cell binding activity of Mitsuba-1

Mitsuba-1 and MytiLec-1 (100 *μ*g/L), after dialysis against 100 mM NaHCO_3_ in saline, were labeled with HiLyte Fluor 555 (Dojindo Molecular Technologies Inc., Kumamoto, Japan) according to the manufacturer’s instructions. Labelled lectin was incubated with Raji cells (5 × 10^5^, in 100 *μ*L culture medium) for 30 min at room temperature. Cells were then washed three times with culture medium, and fluorescence was observed with a BZ-X700 microscope (Keyence Corporation, Osaka, Japan) using 555 nm (excitation) and 570 nm (emission).

### Cell viability assay

Raji cells were maintained in RPMI 1640 medium supplemented with heat-inactivated fetal calf serum 10% (v/v), penicillin (100 IU/ml), and streptomycin (100 *μ*g/mL) at 310 K in an atmosphere of 95% air/5% CO_2_. Cytotoxic activity and cell growth were determined using Cell Counting Kit-8 containing WST-8 (Dojindo Molecular Technologies Inc., Kumamoto, Japan)^56, 57^. Cells (2 × 10^4^, in 90 *μ*L solution) were seeded into a 96-well flat-bottom plate and treated with various concentrations of the recombinant MytiLec-1 or Mitsuba-1 (10 *μ*L of 0–50 *μ*g/mL) for 24 h at 310 K. The effect on cell growth was assayed by addition of WST-8 solution (10 *μ*L) to each well and incubation for 4 h at 310 K. The reduction in the proportion of living cells was assayed by measurement of absorbance at 450 nm (relative to reference absorbance at 600 nm) using the GLOMAX Multi Detection System (Promega, Madison, WI, USA). Results of experiments are presented as the mean ± standard error. Differences in means were evaluated by two-tailed Student’s *t*-test with P values <0.05.

### Isothermal titration calorimetry

ITC experiments were carried out with a MicroCal VP-ITC (Malvern). 20 *μ*M Mitsuba-1 (in 10 mM HEPES pH 7.4, 100 mM NaCl) was placed in the cell, and maintained at a temperature of 298 K. NAcGal was dissolved in the same buffer to a final concentration of 12 mM. 22 injections of this ligand solution, 10 *μ*L each, were made in total, allowing the baseline to stabilise between injections. The raw data were fitted to a single site model using the manufacturer’s software.

## Electronic supplementary material

Supplementary Information
