# Supplementary material for: Computational design of a symmetrical β-trefoil lectin with cancer cell binding activity
Source: Sci Rep. 2017 Jul 19;7:5943. doi: 10.1038/s41598-017-06332-7 (PMC5517649; doi:10.1038/s41598-017-06332-7)
Supplement: Supplementary file 1 — Supplementary Information [file 41598_2017_6332_MOESM1_ESM.pdf]

Computational design of a symmetrical β-trefoil lectin with cancer cell binding activity.

Daiki Terada[1, 2], Arnout R. D. Voet[3], Hiroki Noguchi[3], Kenichi Kamata[1], Mio Ohki[1], Christine Addy[1], Yuki Fujii[4], Daiki Yamamoto[5], Yasuhiro Ozeki[5], Jeremy R. H. Tame[1,*], and Kam Y. J. Zhang[2,*]

[1]Graduate School of Medical Life Science, Yokohama City University, 1-7-29 Suehiro, Yokohama, Kanagawa 230-0045, Japan
[2]Structural Bioinformatics Team, Division of Structural and Synthetic Biology, Center for Life Science Technologies, RIKEN, 1-7-22 Suehiro, Tsurumi, Yokohama, Kanagawa 230-0045, Japan
[3]Laboratory of Biomolecular Modelling and Design, Department of Chemistry, KU Leuven, Celestijnenlaan 200G 3001, Heverlee, Belgium
[4]Department of Pharmacy, Graduate School of Pharmaceutical Science, Nagasaki International University, 2825-7 Huis Ten Bosch, Sasebo, Nagasaki 859-3298, Japan
[5]Laboratory of Glycobiology and Marine Biochemistry, Graduate School of NanoBio Sciences, Yokohama City University, 22-2, Seto, Yokohama, Kanagawa 236-0027, Japan
[*]jtame@tsurumi.yokohama-cu.ac.jp, kamzhang@riken.jp

Supplementary Information

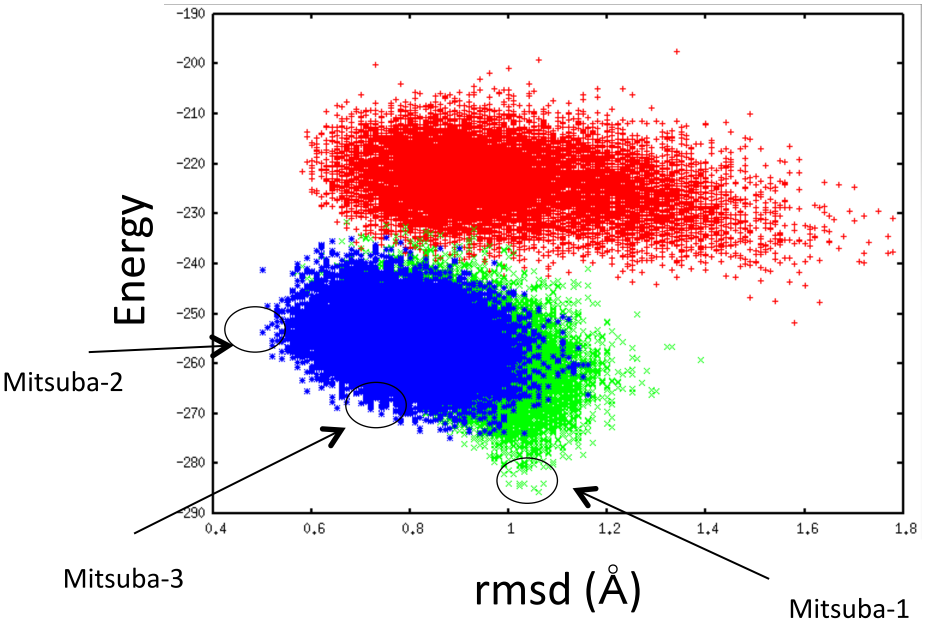

Figure S1. A plot of energy score *vs.* RMSD from the initial backbone structure of different models created by decorating the main-chain with amino acid sequences. Red points indicate models built by sequence decoration of a backbone model created solely from subdomain-A of MytiLec-1. Green points indicate models whose initial backbone was created by incorporating YYK and VDL tripeptides (taken from Threefoil) at the start and end of each subdomain. Blue points indicate models that included YYK and WRLVDL peptides from Threefoil, and both Mitsuba-2 and Mitsuba-3 were selected from this group. Mitsuba-1 includes only the YYK and VDL tripeptides.

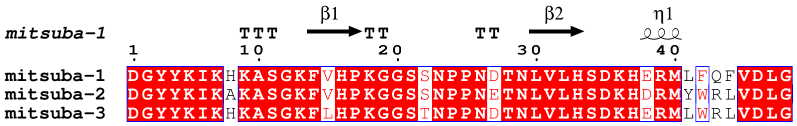

Figure S2. An alignment of the sequences of Mitsuba-1, -2 and -3. The structural elements of Mitsuba-1 are shown as coils and arrows to indicate helices and sheets respectively. The three sequences share 39 out of 48 residues. Notably Mitsuba-1 does not have the tryptophan of the other sequences. The YYK and WRLVDL motifs in Mitsuba-2 and Mitsuba-3 derive from Threefoil. This figure was made with ESPRIPT.[1]

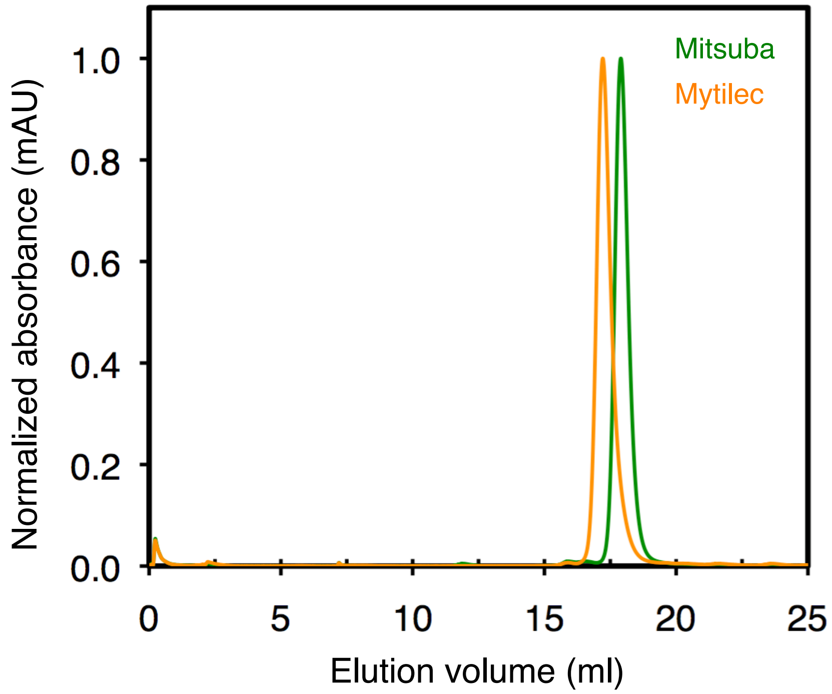

Figure S3. Analytical size-exclusion chromatogram of purified Mitsuba-1 and MytiLec-1. MytiLec-1 is dimeric. The small but significant shift in elution volume results from loss of the dimer interface in Mitsuba-1, making it monomeric.

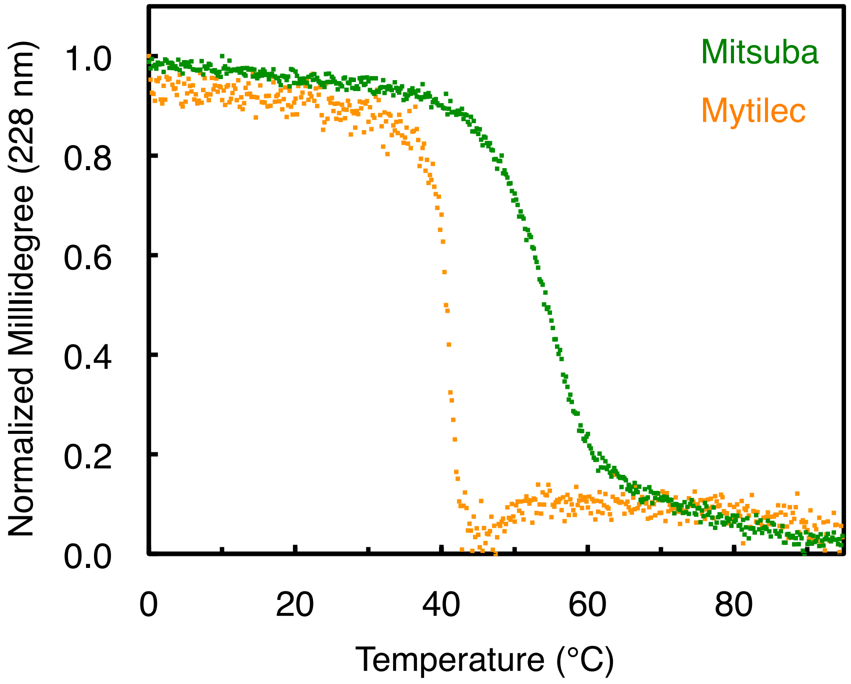

(a) Determination of melting temperatures (T_M) of Mitsuba-1 and MytiLec-1, using circular dichroism to monitor the loss of tertiary structure. MytiLec-1 (orange dots) shows complete unfolding before 50°C is reached, but Mitsuba-1 (green dots) has a T_M of 55°C. The sharper drop in structure of MytiLec-1 is probably due to the dimer dissociating and monomers unfolding in a highly cooperative process, whereas Mitsuba-1 is monomeric.

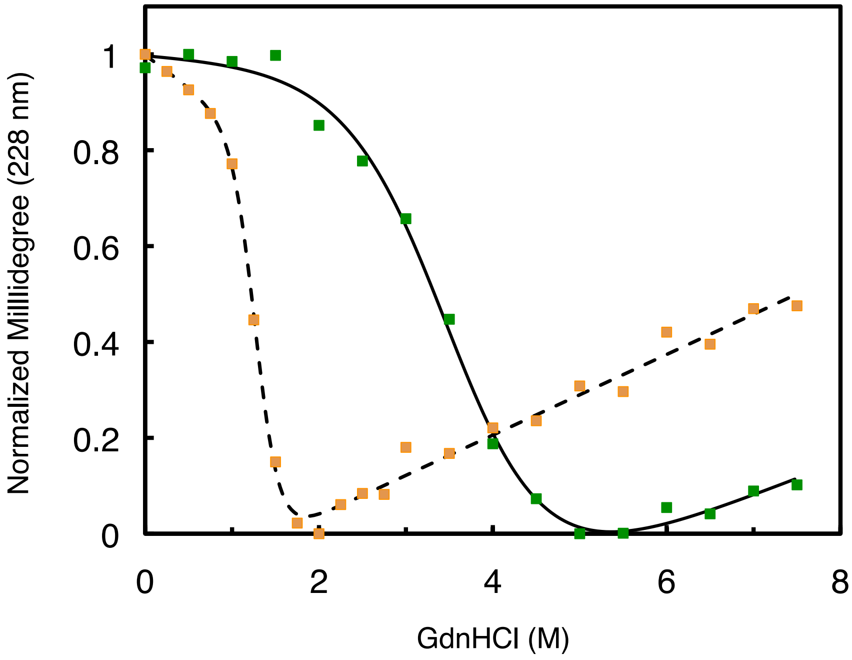

(b) Unfolding Mitsuba-1 and MytiLec-1 with guanidinium hydrochloride, monitored by circular dichroism. MytiLec-1 (orange dots) is completely unfolded by less than 2 molar GdnHCl, but Mitsuba-1 (green dots) retains substantial structure at 3 molar.

Figure S4. Unfolding studies of Mitsuba-1.

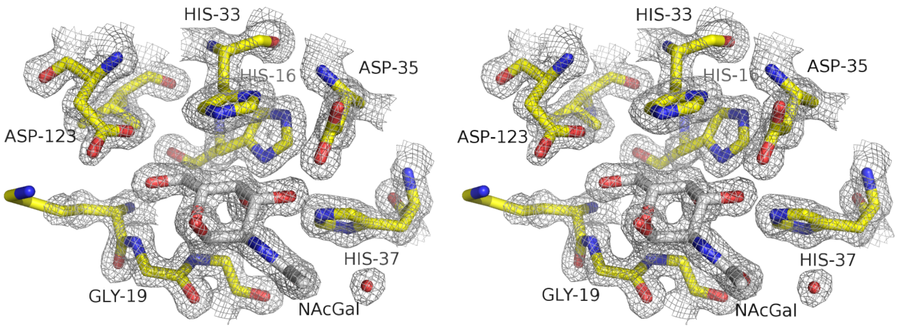

Figure S5. A stereo view of the 2mFo-DFc electron density map, contoured at 1.5 σ, covering one of the sugar ligands.

References

1. Robert, X. & Gouet, P. Deciphering key features in protein structures with the new ENDscript server. *Nucleic Acids Res.* **42**, W320–324 (2014).
